# Simple Spectrophotometric Method for Determination of Paroxetine in Tablets Using 1,2-Naphthoquinone-4-Sulphonate as a Chromogenic Reagent

**DOI:** 10.1155/2009/237601

**Published:** 2009-04-22

**Authors:** Ibrahim A. Darwish, Heba H. Abdine, Sawsan M. Amer, Lama I. Al-Rayes

**Affiliations:** Department of Pharmaceutical Chemistry, College of Pharmacy, King Saud University, P.O. Box 2457, Riyadh 11451, Saudi Arabia

## Abstract

Simple and rapid spectrophotometric method has been developed and validated for the determination of paroxetine (PRX) in tablets. The proposed method was based on nucleophilic substitution reaction of PRX with 1,2-naphthoquinone-4-sulphonate (NQS) in an alkaline medium to form an orange-colored product of maximum absorption peak (*λ*_max_) at 488 nm. The stoichiometry and kinetics of the reaction were studied, and the reaction mechanism was postulated. Under the optimized reaction conditions, Beer's law correlating the absorbance (A) with PRX concentration (C) was obeyed in the range of 1–8 *μ*g mL^−1^. The regression equation for the calibration data was: A = 0.0031 + 0.1609 C, with good correlation coefficients (0.9992). The molar absorptivity (*ε*) was 5.9 × 10^5^ L mol^−1^ 1 cm^−1^. The limits of detection and quantitation were 0.3 and 0.8 *μ*g mL^−1^, respectively. The precision of the method was satisfactory; the values of relative standard deviations did not exceed 2%. The proposed method was successfully applied to the determination of PRX in its pharmaceutical tablets with good accuracy and precisions; the label claim percentage was 97.17 ± 1.06 %. The results obtained by the proposed method were comparable with those obtained by the official method.

## 1. Introduction

Paroxetine; (3*S*,4*R*)-3-[(1,3-benzodioxol-5-vloxy)methyl]-4-(4-flurophenyl) piperidine (PRX) is a new generation antidepressant drug. It exerts its antidepressant effect through a selective inhibition for the reuptake of the neurotransmitter serotonin by the presynaptic receptors. PRX is comparable to the tricyclic antidepressants in their clinical efficacy, however, PRX is safer and has greater acceptance by the patients [1]. It is also prescribed in the treatment of related disorders, such as obsessive-compulsive disorder, panic fits, social phobia, and posttraumatic stress [2]. PRX is devoid of sedative effect and remarkably safe in overdose. PRX takes 5.2 hours to reach the peak, with extended half-life (21 hours) that allowed the introduction of formulations for once-daily dosing [3]. These combined qualities made PRX the most widely prescribed antidepressants [4].

The methods reported for quantitative determination of PRX in tablets and/or biological fluids include voltammetry [5, 6], densitometry [7, 8], high-performance liquid chromatography [9–14], gas chromatography [15–17], and capillary electrophoresis [18]. These methods offered the required sensitivity and selectivity for the analysis of PRX in biological fluids; however, their sophisticated instrumentation and high analysis cost limited their routine use in quality control laboratories for analysis of PRX in its pharmaceutical tablets.

Spectrophotometry is considered the most convenient analytical technique because of its inherent simplicity, low cost, and wide availability in most quality control laboratories. However, few spectrophotometric methods have been reported for its determination in tablets [19–21]. These methods were based on formation of ion-pair associates with bromophenol blue, bromothymol blue, and bromocresol green [19], formation of charge-transfer complexes with 7,7,8,8-tetracyanoquinodimethane and chloranilic acid [20], formation of vinylamino-substituted haloquinone derivatives with haloquinone reagents in presence of acetaldehyde [21], and formation of condensation product with 7-chloro-4-nitrobenzofurazon [19]. These methods were associated with some major drawbacks such as laborious multiple extraction steps in the analysis by ion pair formation-based methods [19], and in preparation of the samples for the analysis by the methods relied on PRX base, rather than the hydrochloride salt [20, 21]. Furthermore, the analytical reactions were long and thus the procedures were time-consuming [19, 21]. For these reasons, the development of new alternative simple and rapid spectrophotometric method for the determination of PRX in its tablets was very essential.

1,2-naphthoquinone-4-sulphonic sulphonate (NQS) has been used as a chromogenic reagent for the spectrophotometric determination of many pharmaceutical amines [22–26]. However, the reaction between NQS and PRX has not been investigated so far. The present study describes the evaluation of NQS as a chromogenic reagent in the development of simple and rapid spectrophotometric method for the determination of PRX in its tablets.

## 2. Experimental

### 2.1. Apparatus

Double beam V-530 (JASCO Co. Ltd., Kyoto, Japan) ultraviolet-visible spectrophotometer with matched 1 cm quartz cells was used for all the spectrophotometric measurements. pH meter, Model 350 (Bibby Scientific Ltd., T/As Jenway, Essex, UK).

### 2.2. Reagents and Materials

Paroxetine hydrochloride (PRX; SmithKline Beecham Pharmaceuticals, Bentford, England) was obtained and used as received; its purity was 99.8 ± 1.45%. A solution of 0.5% (w/v) of 1,2-naphthoquinone-4-sulphonate (NQS; Aldrich Chemical Co., St. Louis, Mo, USA) was prepared by dissolving 250 mg in 50 mL distilled water. The solution was freshly prepared and protected from light during use. Clark and Lubs buffer solution of pH 9 was prepared by mixing 50 mL of 0.2 M aqueous solution of boric acid and potassium chloride (1 liter contains 12.368 g of boric acid and 14.90 g of potassium chloride) with 21.3 mL of 0.2 M sodium hydroxide in 200 mL standard flask [27] and adjusted by pH meter. Seroxate tablets (SmithKline Beecham Pharmaceuticals, Brentford, UK) are labeled to contain 20 mg paroxetine HCl per tablet. Double distilled water was obtained through WSC-85 water purification system (Hamilton Laboratory Glass Ltd., Ky, USA) and used throughout the work. All solvents and materials used throughout this study were of analytical grade.

### 2.3. Preparation of Standard and Sample Solutions

#### 2.3.1. Paroxetine Hydrochloride (PRX) Standard Solution

An accurately weighed amount (50 mg) of PRX was quantitatively transferred into a 25 mL calibrated flask, dissolved in 20 mL distilled water, completed to volume with the same solvent to obtain a stock solution of 2 mg mL^−1^. This stock solution was further diluted with water to obtain working solutions in the range of 10–80 *μ*g mL^−1^.

#### 2.3.2. Tablets Sample Solution

Twenty tablets were weighed and finely powdered. An accurately weighed quantity of the powdered tablets equivalent to 100 mg of PRX was transferred into a 100 mL calibrated flask and dissolved in about 40 mL of distilled water. The contents of the flask were swirled, sonicated for 5 minutes, and then completed to volume with water. The contents were mixed well and filtered rejecting the first portion of the filtrate. The prepared solution was diluted quantitatively with distilled water to obtain a suitable concentration for the analysis.

### 2.4. General Recommended Procedure

Accurately measured aliquots of PRX solution containing 10–50 *μ*g mL^−1^ were transferred into separate 10 mL calibrated flasks. One milliliter of Clark and Lubs buffer solution (pH 9) was added followed by 1 mL of NQS solution (0.5%, w/v). The reaction solution was allowed to proceed at room temperature (25 ± 5°C) for 10 minutes. The reaction mixture was completed to volume with methanol, and the resulting solution was measured at 488 nm against reagent blank treated similarly.

### 2.5. Determination of the Stoichiometric Ratio of the Reaction

#### 2.5.1. Job's Method

Job's method of continuous variation [28] was employed. Master equimolar (5 × 10^−3^ M) aqueous solutions of PRX and NQS were prepared. Series of 10 mL portions of the master solutions of PRX and NQS were made up comprising different complementary proportions (0 : 10, 1 : 9, …, 9 : 1, 10 : 0, inclusive) in 10 mL calibrated flasks containing 1 mL of buffer solution (pH 9). The solution was manipulated as described under the general recommended procedures, Section 2.4.

#### 2.5.2. Limiting Logarithmic Method

In the limiting logarithmic method [29], two sets of experiments were carried out employing the general recommended procedures described above. The first set of experiments was carried out using increasing NQS concentrations (1.9 × 10^−3^ − 9.6 × 10^−3^ M) at fixed PRX concentration (1.37 × 10^−5^ M). The second set of experiments was carried out using increasing PRX concentrations (0.3 × 10^−5^ − 2.04 × 10^−5^ M) at fixed NQS concentration (1.92 × 10^−2^ M). The logarithms of the obtained absorbances were plotted as function of the logarithms of the NQS and PRX concentration in the first and second sets of experiments, respectively. The slopes of the fitting lines in both sets of experiments were calculated.

## 3. Results and Discussion

### 3.1. Absorption Spectra

**Figure 1:**
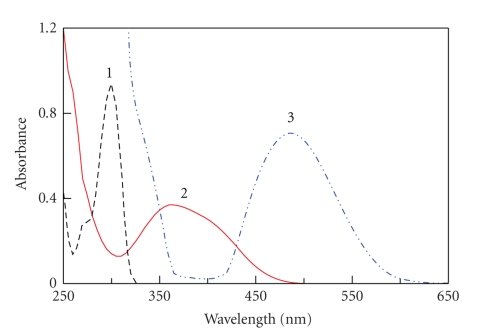
(1) Absorption spectra of PRX against water, (2) NQS against water, and (3) their reaction product against reagent blank.

According to the procedure, the absorption spectrum of the product produced by the reaction between PRX and NQS was recorded (Figure 1). The product was orange-colored exhibiting a maximum absorption peak (*λ*_max _) at 488 nm, and the *λ*_max _ of NQS was 360 nm. The *λ*_max _ of the product was red-shifted by 248 nm from the *λ*_max _ of RRX (240 nm). In order to eliminate the interference, the measurements were carried out at 488 nm against the reagent blank.

### 3.2. Optimization of Reaction Variables

#### 3.2.1. Effect of NQS Concentration

**Figure 2:**
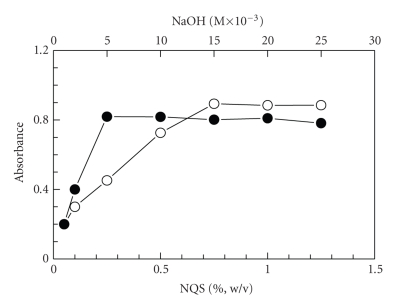
Effect of NQS (•) and NaOH (∘) concentrations on the reaction of PRX with NQS. PRX (40 *μ*g mL^−1^): 1 mL; NaOH: 1 mL; NQS: 1 mL; temperature: 25 ± 5°C; reaction time: 10 minutes.

The studying of NQS concentrations revealed that the reaction was dependent on NQS reagent (Figure 2). The absorbance of the reaction solution increased as the NQS concentration increased, and the highest absorption intensity was attained at NQS concentration of 0.25% (w/v). Higher NQS concentrations up to 1.25% had no effect on the absorption values. Further experiments were carried out using 0.5%.

#### 3.2.2. Effect of Alkalinity and pH

**Figure 3:**
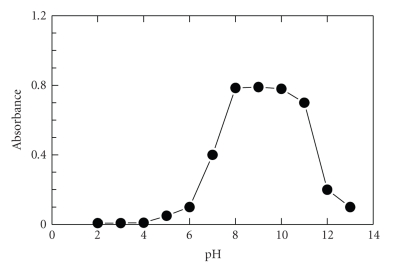
Effect of pH on the reaction of PRX with NQS. PRX (40 *μ*g mL^−1^): 1 mL; Clark and Lubs buffer solution: 1 mL; NQS (0.5%, w/v): 1 mL; temperature: 25 ± 5°C; reaction time: 10 minutes.

To generate the nucleophile from PRX and activate the nucleophilic substitution reaction, alkaline medium was necessary. Different inorganic bases were tested: sodium hydroxide, disodium hydrogen phosphate, and sodium bicarbonate, all prepared as aqueous solution of a concentration range of 1–25 × 10^−3^ M. Best results were obtained in case of sodium hydroxide where with other bases either precipitation of white colloid occurred upon diluting the reaction solution with organic solvent, high blank readings, nonreproducible results, and/or weak sensitivity were observed. Studies for optimization of sodium hydroxide concentration revealed that the optimum concentration was 2–25 × 10^−3^ M (Figure 2). As well, it was found that the use of alkaline buffer solution gives more precise readings over the use of NaOH. In a separate series of experiments, the influence of pH on the absorbance of PRX-NQS product was investigated. The results revealed that the absorbances at pH < 6 were close to 0, indicating that under acidity, PRX has difficulty to react with NQS (Figure 3). This was possibly due to the fact that the amino group (piperazinyl-NH) of PRX exists in the form of hydrochloride amine salt, thus, it loses the nucleophilic substitution capability. At pH > 6, the absorbance increased rapidly with the increase in the pH, as the amino group of PRX turns into the free-NH, rather than the HCl salt, facilitating the nucleophilic substitution reaction. The maximum absorption values were attained in the range of pH at 8–10. At pH > 10, the absorbance of solution obviously decreased. This was attributed probably to the increase in the amount of hydroxide ion that holds back the condensation reaction between PRX and NQS. In order to keep the high sensibility for determination of PRX, the experiment was carried out at pH 9.

#### 3.2.3. Effect of Temperature and Time

**Figure 4:**
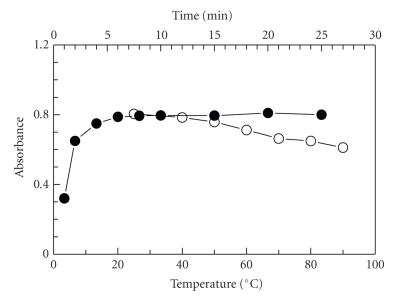
Effect of temperature (∘) and time (•) on the reaction of PRX with NQS. PRX (40 *μ*g mL^−1^): 1 mL; Clark and Lubs buffer solution (pH 9): 1 mL; NQS (0.5%, w/v): 1 mL.

The effect of temperature on the reaction was studied by carrying out the reaction at different temperatures (25–90°C). The results (Figure 4) revealed that increasing the temperature had negative effect on the absorption values of the reaction solution. This was probably attributed to the instability of the PRX-NQS derivative. For this reason, further experiments were carried out at room temperature (25 ± 5°C). The effect of time on the formation of the reaction product was investigated by carrying out the reaction for different times. The maximum absorbance intensity was attained after 5 minutes, and longer reaction time up to 25 minutes did not affect the absorbance intensity (Figure 4). For more precise results, further experiments were carried out at 10 minutes.

#### 3.2.4. Effect of Organic Solvents

It was found that the PRX-NQS product is insoluble in the aqueous reaction medium. For spectrophotometric measurements, the reaction product might be either dissolved in a miscible organic solvent of lower polarity than water or extracted with an immiscible extractive solvent. Different solvents were tested for dilution: methanol, ethanol, isopropanol, acetone, acetonitrile, dimethylsulphoxide, and 1,4-dioxane. The highest readings were obtained when methanol was used for dilution (Table 1). In a separate series of experiments, different nonmiscible solvents were tested for extraction of the PRX-NQS product: carbon tetrachloride, chloroform, dichloromethane, ethyl acetate, toluene, and benzene. The highest readings were obtained when chloroform was used for extraction. The performance of both extractive and nonextractive procedures (in terms of sensitivity and background readings) was comparable. In order to simplify the analytical procedures, the simple nonextractive procedure (dilution with methanol) was chosen as optimum condition for the further experiments.

#### 3.2.5. Stability of the Chromogen

Under the aforementioned optimum conditions, the reaction between PRX and NQS was completed within 5 minutes at room temperature, and the absorbance no longer changed after standing for up to 25 minutes. The effect of time on the stability of the chromogen was studied by following the absorption intensity of the reaction solution (after dilution) at different time intervals. It was found that the absorbance of the chromogen remains stable for at least 4 hours. This allowed the processing of large batches of samples and their comfortable measurements with convenience. This increased the convenience of the methods as well as made it applicable for large number of samples.

### 3.3. Stoichiometry and Kinetics of the Reaction

**Figure 5:**
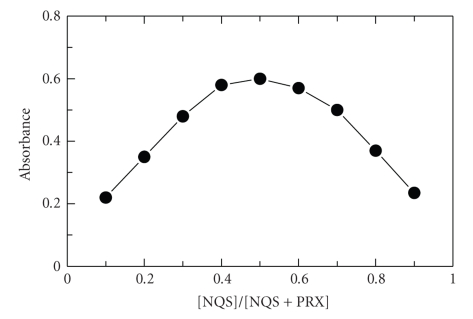
Job's plot for determination of stoichiometry of the reaction between PRX and NQS. [PRX]: 5 × 10^−3^ M; [NQS]: 5 × 10^−3^ M; [PRX]+[NQS]: 1 mL; Clark and Lubs buffer solution (pH 9): 1 mL; temperature: 25 ± 5°C; reaction time: 10 minutes.

**Figure 6:**
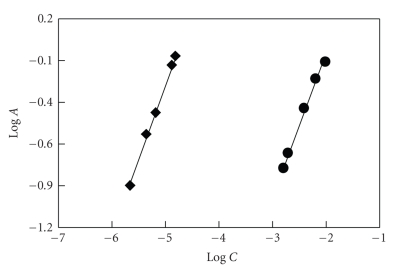
Limiting logarithmic plot for molar reactivity of PRX with NQS. *C* and *A* are the concentration and absorbance, respectively. For generating the first line (◆), [NQS]: 1.9 × 10^−2^; [PRX]: 0.3 × 10^−5^ − 2.04 × 10^−5^ M; Clark and Lubs buffer solution (pH 9): 1 mL; temperature: 25 ± 5°C; reaction time: 10 minutes. For generating the second line (•), [NQS]: 1.9 × 10^−3^ − 1.9 × 10^ −2^ M; [PRX]: 1.37 × 10^−5^ M. The other conditions are the same as those employed in generating the first line.

**Figure 7:**
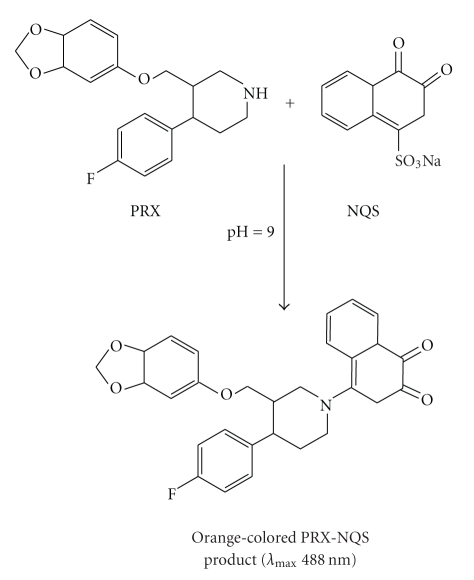
Scheme for the reaction pathway of PRX with NQS.

Under the optimum conditions, the stoichiometry of the reaction between PRX and NQS was investigated by Job [28] and limiting logarithmic [29] methods. The symmetrical bell shape of Job's plot (Figure 5) indicates that the NQS:PRX ratio was 1 : 1. In the limiting logarithmic method, two straight lines were obtained (Figure 6). The values of the slopes of these lines were 1.0201 and 0.9248, confirming the 1 : 1 ratio for the reaction. Based on this ratio, and the presence of only one center (piperazinyl N–H group) in PRX molecule that is available for the substitution reaction, the reaction pathway was postulated to be proceeded as shown in Figure 7.

**Figure 8:**
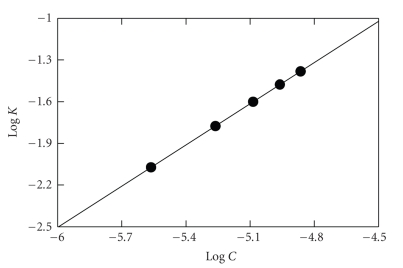
Linear plot for Log *C* versus Log *K* for the kinetic reaction of PRX with NQS. *C* is the PRX concentration (0.3 × 10^−5^ − 2.04 × 10^−5^ M) and *K* is the reaction rate (second^−1^). Clark and Lubs buffer solution (pH 9): 1 mL; NQS (1.9 × 10^−2^): 1 mL; temperature: 25 ± 5°C.

Under the optimum conditions, the absorbance-time curves for the reaction of PRX at several concentrations (0.3 × 10^−5^ − 2.04 × 10^−5^ M) with a fixed concentration of NQS (1.9 × 10^−2^ M) were generated, and the initial reaction rates (*K*) were determined from the slopes of the curves. The logarithms of the reaction rates (Log *K*) were plotted as a function of logarithms of PRX concentration (log *C*). As seen in Figure 8, a straight line passing through the origin with a slope value of 0.9888 was obtained by fitting the data to the following equation:

(1) $$\text{Log }K=\text{log }K^{\prime }+n\;\text{ log }C,$$

where *K* is reaction rate, *K*′ is the rate constant, *C* is the molar concentration of PRX, and *n* (slope of regression line) is the order of the reaction. The value of the slope (≈1) confirmed that the reaction was first order. However, under the optimized reaction conditions, the concentration of NQS was in much more excess than that of PRX in the reaction solution. Therefore, the reaction was regarded as a pseudo-first-order reaction.

### 3.4. The Apparent Rate Constant and Activation Energy

The absorbance-time curves at three different temperatures (25, 40, and 60°C) were generated using fixed concentrations of PRX (1.7 × 10^−5^ M) and NQS (1.9 × 10^−2^ M). From these curves, the apparent rate constants were calculated. These rates were found to be 6.92 × 10^−4^, 6.7 × 10^−4^, and 6.25 × 10^−4^ second^−1^ at 25, 40, and 60°C, respectively. The activation energy, defined as the minimum kinetic energy that a molecule possess in order to undergo a reaction, was determined using Arrhenius equation [30]:

(2) Log  k=log  A−Ea/2.303   RT,

where *k* is the apparent rate constant, *A* is the frequency factor, Ea is the activation energy, *T* is the absolute temperature, and *R* is the gas constant. By plotting log *k*′ as a function of 1/*T*, a straight line with a slope value of 1.775 = −Ea /2.303 *R*. From this data, the activation energy was found to be 8.12 kcal mole^−1^. Because of this low activation energy, the nucleophilic substitution reaction between PRX and NQS could be easily taken place, and NQS could be used for determination of PRX.

### 3.5. Validation of the Method

#### 3.5.1. Calibration and Sensitivity

Calibration curve for the determination of PRX by its reaction with NQS was constructed by plotting the absorbances as a function of the corresponding concentrations. The regression equation for the results was *A* = 0.0031 + 0.1609*C* (*r* = 0.9992), where *A* is the absorbance at 488 nm, *C* is the concentration of PRX in *μ*g mL^−1^ in the range of 1–8 *μ*g mL^−1^, and *r* is the correlation coefficient. The molar absorptivity (*ε*) was 5.9 × 10^5^ L mol^−1^ cm^−1^. The limit of detection (LOD) and limit of quantitation (LOQ) were determined using the formula: LOD or LOQ = *κ*SDa/b, where *κ* = 3 for LOD and 10 for LOQ, SDa is the standard deviation of the intercept, and b is the slope. The LOD and LOQ were 0.3 and 0.8 *μ*g mL^−1^, respectively. The precision of the proposed method was determined by analyzing 5 replicate samples of standard PRX solution at one concentration level. The assay gave satisfactory results; the relative standard deviation (RSD) was less than 2%.

#### 3.5.2. Reproducibility

The reproducibility of the proposed method was determined by replicate analysis of five separate solutions of the working standard at three concentration levels of each drug (1.5, 3, and 6 *μ*g mL^−1^). The method gave satisfactory results; RSD did not exceed 2% indicating the good reproducibility of the proposed method. This precision level is adequate for the precision and routine analysis of the investigated drugs in quality control laboratories.

#### 3.5.3. Accuracy and Interference Liabilities

The accuracy of the proposed method was evaluated by the standard addition method. The recovery values of the added concentrations were 97.6 − 101.3 ± 0.84 − 1.85% (Table 2), indicating the accuracy of the proposed method. Before proceeding with the analysis of PRX in its tablets, interference liabilities were carried out to explore the effect of common excipients that might be added during tablets formulation. Samples were prepared by mixing known amount (20 mg) of PRX with various amounts of the common excipients: starch, glucose, lactose, acacia, talc, and magnesium stearate. These laboratory-prepared samples were analyzed by the proposed method applying the general recommended procedure. The recovery values were 97.97 − 101.53 ± 0.39 − 1.26%, with an average recovery of 99.63 ± 1.23% (Table 3). These data confirmed the absence of interference from any of the common excipients with the determination of PRX by the proposed method.

#### 3.5.4. Robustness and Ruggedness

Robustness was examined by evaluating the influence of small variation of method variables including concentration of analytical reagent and reaction time on the performance of the proposed methods. In these experiments, one parameter was changed whereas the others were kept unchanged, and the recovery percentage was calculated each time. It was found that small variation of method variables did not significantly affect the procedures; recovery values were 98.8 − 101.5 ± 0.85 − 1.87% (Table 4). This provided an indication for the reliability of the proposed method during its routine application for the analysis of PRX. Ruggedness was also tested by applying the proposed methods to the assay of PRX using the same operational conditions but using two different instruments at two different laboratories and different elapsed time. Results obtained from lab-to-lab and day-to-day variations were reproducible, as the relative standard deviations (RSDs) did not exceed 2.54%.

### 3.6. Application of the Proposed Method to Analysis of PRX in Tablets

It is evident from the above-mentioned results that the proposed method gave satisfactory results with PRX in bulk. Thus, its tablets were subjected to the analysis of their PRX contents by the proposed and the official [14] methods. The label-claim percentage was 99.17 ± 1.06% (Table 5). This result was compared with that obtained from the official method by statistical analysis with respect to the accuracy (by *t*-test) and precision (by *F*-test). No significant differences were found between the calculated and theoretical values of *t*- and *F*-tests at 95% confidence level proving similar accuracy and precision in the determination of PRX by both methods.

## 4. Conclusions

The present study described the successful evaluation of NQS reagent in the development of simple and rapid spectrophotometric method for the accurate determination of PRX in bulk and tablets. In contrast with the previously reported methods for analysis of PRX, the method described herein has many advantages: it does not need expensive sophisticated apparatus, it is simple and rapid, and it has high sensitivity. Furthermore, all the analytical reagents are inexpensive, have excellent shelf life, and are available in any analytical laboratory. Therefore, this method is practical and valuable for its routine application in the analysis of PRX in quality control laboratories.

## Figures and Tables

**Table 1:** Effect of diluting and extracting solvents on the intensity of the reaction product of PRX with NQS.

Diluting solvent	Absorbance^(a)^	Extracting solvent	Absorbance^(a)^
Methanol	0.802	Carbon tetrachloride	0.587
Ethanol	0.781	Chloroform	0.808
Isopropanol	0.778	Dichloromethane	0.716
Acetone	0.245	Ethyl acetate	0.362
Acetonitrile	0.686	Toluene	0.267
Dimethylformamide	0.346	Benzene	0.239
1,4-dioxane	0.617		

^(a)^Values for all solvents are mean of three determinations; the RSDs for the readings were <3.

**Table 2:** Recovery studies for determination of PRX by the proposed method.

Sample number	PRX		Recovery (% ± SD)^(a)^
	Added (*μ*g mL^−1^)	Found (*μ*g mL^−1^)^(a)^	
1	2.0	1.99	99.5 ± 1.85
2	3.0	2.97	98.9 ± 1.20
3	4.0	4.05	101.3 ± 0.92
4	5.0	4.88	97.6 ± 0.84
5	6.0	5.98	99.7 ± 1.50

^(a)^Values are mean of three determinations.

**Table 3:** Analysis of PRX in presence of common excipients by the proposed method.

Excipient	Recovery (% ± SD)^(a)^
Starch (50)^(b)^	99.46 ± 0.46
Glucose (10)	99.58 ± 1.23
Lactose (10)	100.37 ± 0.87
Acacia (10)	98.84 ± 0.39
Talc (5)	101.53 ± 1.02
MS^(c)^(10)	97.97 ± 1.26

Average ± SD	99.63 ± 1.23

^(a)^Values are mean of three determinations.

^(b)^Figures in parenthesis are the amounts in mg added per 20 mg of PRX.

^(c)^MS: Magnesium stearate.

**Table 4:** Influence of small variations in the assay conditions on the analytical performance of the proposed spectrophotometric method for determination of PRX using NQS reagent.

Parameters	Recovery (% ± SD)^(a)^
Recommended conditions^(b)^	100.5 ± 1.35
NQS concentration (%, w/v)	
0.25	98.8 ± 0.85
0.75	100.4 ± 1.25
Buffer solution (pH)	
8.8	98.9 ± 1.87
9.2	101.5 ± 1.67
Reaction time (min)	
5	99.5 ± 1.87
15	100.2 ± 0.85

^(a)^Values are mean of 3 determinations.

^(b)^The recommended conditions are given in Section 2.

**Table 5:** Analysis of PRX-containing tablets by the proposed and the official methods.

Tablet	Recovery (% ± RSD)^(a)^		*t*-value^(c)^	*F*-value^(c)^
	Proposed	Official^(b)^		
Seroxate tablets	99.17 ± 1.06	101.31 ± 0.48	2.20	4.88

^(a)^Values are mean of 6 determinations.

^(b)^Reference [14].

^(c)^The tabulated values of *t*- and *F*-values at 95% confidence limit are 2.78 and 6.39, respectively.
